# Covid-19 telescreening in SUS users with risk conditions: case report

**DOI:** 10.11606/s1518-8787.2020054002953

**Published:** 2020-10-20

**Authors:** Daniela Arruda Soares, Danielle Souto Medeiros, Clavdia Nicolaevna Kochergin, Matheus Lopes Cortes, Sostenes Mistro, Márcio Galvão Oliveira, José Andrade Louzado, Vanessa Moraes Bezerra, Edson Amaro, Hélio Penna Guimarães, Juliede Rosa da Silva, Maria Tânia Silva Oliveira, Jéssica de Oliveira Sousa, Vivian Carla Honorato dos Santos de Carvalho

**Affiliations:** I Universidade Federal da Bahia Instituto Multidisciplinar em Saúde Programa de Pós-Graduação em Saúde Coletiva Vitória da ConquistaBA Brasil Universidade Federal da Bahia. Instituto Multidisciplinar em Saúde. Programa de Pós-Graduação em Saúde Coletiva. Vitória da Conquista, BA, Brasil; II Universidade Federal da Bahia Instituto Multidisciplinar em Saúde Núcleo de Cuidado Integral e Assistência Especializada Vitória da ConquistaBA Brasil Universidade Federal da Bahia. Instituto Multidisciplinar em Saúde. Núcleo de Cuidado Integral e Assistência Especializada. Vitória da Conquista, BA, Brasil; III Hospital Israelita Albert Einstein São PauloSP Brasil Hospital Israelita Albert Einstein. São Paulo, SP, Brasil; IV Prefeitura Municipal de Vitória da Conquista Secretaria de Saúde Vitória da ConquistaBA Brasil Prefeitura Municipal de Vitória da Conquista. Secretaria de Saúde. Vitória da Conquista, BA, Brasil

**Keywords:** Coronavirus Infections, diagnosis, Telemedicine, Students, Medical, Unified Health System

## Abstract

This case report aims to describe the conception and preliminary data of the implementation of a telescreening and telemonitoring program of covid-19 for users of the Unified Health System with risk conditions. A system of telerscreening was implemented through which undergraduate students in the health area contact patients by telephone, according to periodicity and predefined criteria, to monitor the evolution of the condition. In eight weeks, 2,190 attempts at remote contact were made with individuals from five health units. The effective number of individuals monitored at the time this writing is 802.

## INTRODUCTION

The emergence of the new coronavirus, called Sars-CoV-2, presents an unprecedented challenge for health professionals worldwide, causing global impacts on health, politics and the economy within a few months. In January 2020, the World Health Organization (WHO) declared covid-19 a public health emergency of international interest and, in March, its pandemic character^1^. Although deaths related to the disease occur mainly among older people, more recent studies indicate that individuals with polymorbidities, such as hypertension, diabetes and cardiovascular diseases, may have a higher risk of complications due to the disease^2^.

Although it has been a widely used strategy to minimize the spread of the disease, social isolation has brought difficulties in ensuring comprehensive care for users’ health, including the continuous search for active cases of covid-19 and their contacts, as recommended by WHO^3^. There are reports that telemedicine has been used to delay the spread of Sars-CoV-2, maintaining social distancing and providing care through telephone services in mild cases, but this modality of care has limitations in urgent cases^4^.

In an effort to maintain covid-19 screening, especially among groups considered at risk, and to continue providing essential care to users, telescreening can be a safe and crucial mechanism for maintaining care, especially among users at higher risk, who may rapidly progress to acute respiratory failure. In this article, we describe the conception and preliminary data of the implementation of a covid-19 telescreening program with users of the Unified Health System (SUS) with risk conditions in Vitória da Conquista, a medium-sized municipality in the state of Bahia, between May 31 and July 30, 2020.

## METHODS

This article presents a case report of the implementation of a telescreening program for patients with flu-like syndrome, belonging to covid-19 risk groups, registered as users of family health units (FHU) in the municipality of Vitória da Conquista, Bahia.

The municipality has an estimated population of 341,597 inhabitants, according to IBGE data from 2019, and it is home to the macro-region of Southwest Bahia, a reference for approximately 80 municipalities^5^. Primary care in the municipality is divided between 47 family health teams, 7 basic health units and 4 expanded family health and primary care centers, with population coverage of 48% by the Family Health Strategy, and 60% for primary care, according to data from the portal e-Gestor AB, of the Ministry of Health^6^. Primary care in the municipality did not use technical health strategies for non-face-to-face care of SUS users before the covid-19 pandemic.

A working group composed of professors from a local university, primary care professionals and representatives of the Primary Care Board of the Municipal Health Department elaborated a telescreening and telemonitoring flow of patients with signs and symptoms of flu-like syndrome. In this flow, the stage of face-to-face care was also included.

The flow aimed at the identification of patients considered at higher risk for worsening of covid-19, such as the older ones, patients with chronic or autoimmune diseases and obese individuals (Figure). The telephone numbers of these users were identified by the e-SUS Primary Care system or provided by community health agents. For telescreening and telemonitoring, telephone lines were rented and made available to undergraduate students in the health area.

**Figure:**
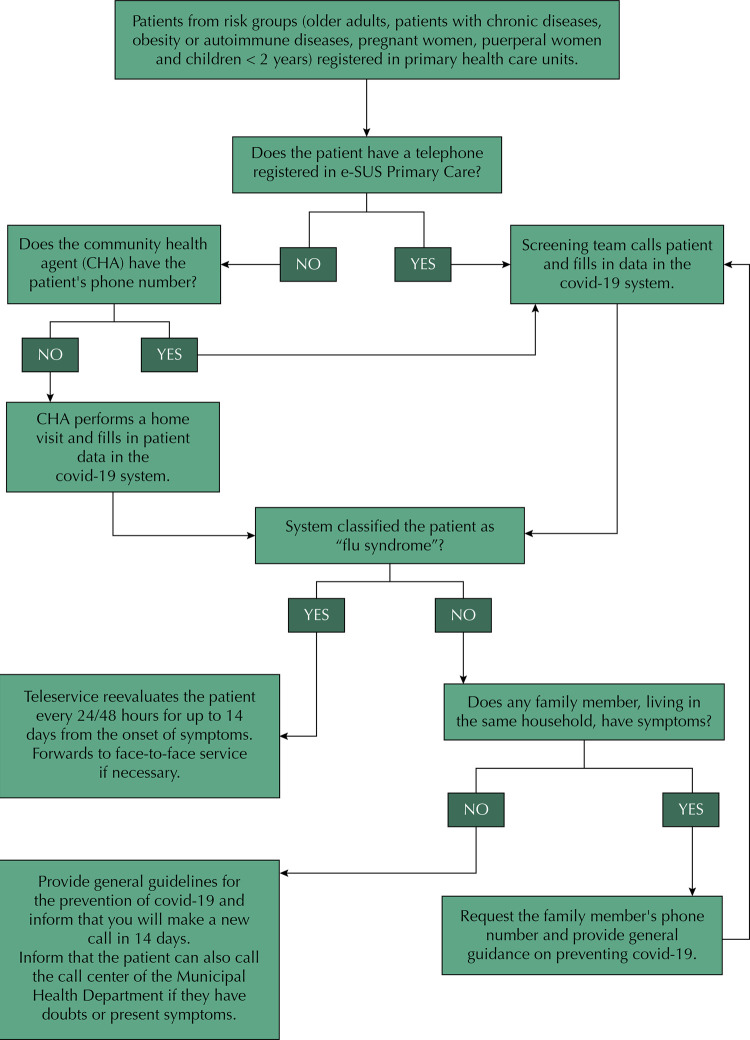
Flowchart of the covid-19 telescreening in primary health care patients with risk of developing a severe condition, in the municipality of *Vitória da Conquista – Bahia*.

Users registered in the FHU received the first call during business hours. At first, the reason for the call was explained and data were collected. Data were released in a system on the web platform for monitoring cases of covid-19, developed by the Information Technology Center of the municipality. This application functions as a clinical decision support system that classifies the user as asymptomatic or with mild or severe flu syndrome, according to the criteria of the Ministry of Health. Individuals with mild influenza syndrome and who have polymorbidities are classified by the system as moderate influenza syndrome, considering the higher risk of worsening covid-19 in these cases.

For cases of mild or moderate flu syndrome, family health teams monitor users every 48 hours, and for cases of severe syndrome, every 24 hours. Asymptomatic cases receive another phone call from the telescreening team within 14 days. In all symptomatic cases, RT-PCR or serological tests are performed by the team of the Municipal Health Department. Serological tests are performed for cases that are not in the optimum material collection time window for RT-PCR. All cases positive for covid-19 and their contacts are monitored by primary care teams, including the epidemiological surveillance of the municipality.

Health teams and students involved in telescreening and telemonitoring received training to use the web platform developed. The Municipal Health Department disclosed the action among the residents of the neighborhoods to increase the community’s support in answering the calls. Data were described using relevant central trend measures and simple frequency.

## RESULTS

After eight weeks, 2,190 attempts to contact the individuals remotely were made. Of this total, the effective number of monitored individuals was 802 (36.6%), with a mean age of 59.7 years (SD=18.5), 532 (66.3%) were women. We identified 15 (1.6%) individuals with mild or moderate flu syndrome and 7 (0.7%) with severe flu-like syndrome, who were referred to teleconsultation. The percentage of telephone calls refused was 38.9% (853).

Another aspect that stood out was the percentage of telephone numbers that were not updated in the municipal health system: 24.4% (535) having justified with the non-obligation of the telephone number for municipal registration in the FHU or the loss of the line due to the patients’ financial conditions. To overcome these challenges, some strategies were included, such as digital cards for dissemination via messaging apps, printed materials, such as posters, disclosure in the territories by community health agents and in the FHU by professionals who provide face-to-face care. For outdated phone numbers, community agents actively searched individuals in the territory and, when possible, performed the screening in person.

## DISCUSSION

Covid-19 screening was not possible in more than half of the users contacted. The data show how primary health care needs to discuss issues related to the use of information and communication technologies within its work routine, understanding that this action will expand the community’s access to health services, not only in the pandemic period. Primary health care should be conducted within the care network, consolidating the use of information and communication technologies and supporting actions to monitor information and strengthen health care^7^.

Functioning as a remote contact device with the patient for symptom screening through specific questions, telescreening is an important tool to promote safe access to users. Its objective is to reduce the time required to obtain a diagnosis, to start treatment and establish the necessary care protocols, as well as to allow home monitoring of users, avoiding the saturation of the units and the risk of contagion, informing citizens and proposing a communication strategy in times of social distancing^4^. This strategy helps primary health care with the home management of users’ health conditions, maintaining longitudinal and continuous care.

Many remote contacts were made in a short period, which helped identify some suspicious patients, later monitored by the health teams of the units. At this time it is possible to provide users with general guidance, in addition to increasing awareness about the pandemic and relieving the psychological stress of the communities monitored. Even through home visits by community agents, it would be difficult to reach all patients, and there is still the advantage of a probable protection of health professionals, without direct contact with patients suspected of covid-19 infection. Moreover, the use of technical health tools can be useful for the routine care of primary care in the post-pandemic period. The new approach also had a low cost and proved useful in monitoring users by community health agents and even by other professionals of the family health teams.

Because it is a case report, the results have some limitations. The reality of a medium-sized municipality may not be reproducible in smaller cities, where the quality of the mobile phone or internet signal presents greater instability. In addition, undergraduate students performed telescreening.

Telescreening proved to be an operationally viable action, promoting rapid communication with the communities, and it should be strengthened for the monitoring of chronic noncommunicable diseases and support to the routine of services. In this pandemic period, telescreening was important for the early care of users.
