# Supplementary material for: Covid-19 telescreening in SUS users with risk conditions: case report
Source: Rev Saude Publica. 2020 Oct 20;54:101. doi: 10.11606/s1518-8787.2020054002953 (PMC7575219; doi:10.11606/s1518-8787.2020054002953)
Supplement: Translation

# Translation: Telerrastreio da covid-19 em usuários do SUS com condições de risco: relato de experiência

**Authors:** Daniela Arruda Soares, Danielle Souto Medeiros, Clavdia Nicolaevna Kochergin, Matheus Lopes Cortes, Sostenes Mistro, Márcio Galvão Oliveira, José Andrade Louzado, Vanessa Moraes Bezerra, Edson Amaro, Hélio Penna Guimarães, Juliede Rosa da Silva, Maria Tânia Silva Oliveira, Jéssica de Oliveira Sousa, Vivian Carla Honorato dos Santos de Carvalho

**Affiliations:** I Brasil Universidade Federal da Bahia. Instituto Multidisciplinar em Saúde. Programa de Pós-Graduação em Saúde Coletiva. Vitória da Conquista, BA, Brasil; II Brasil Universidade Federal da Bahia. Instituto Multidisciplinar em Saúde. Núcleo de Cuidado Integral e Assistência Especializada. Vitória da Conquista, BA, Brasil; III Brasil Hospital Israelita Albert Einstein. São Paulo, SP, Brasil; IV Brasil Prefeitura Municipal de Vitória da Conquista. Secretaria de Saúde. Vitória da Conquista, BA, Brasil

**Keywords:** Coronavirus Infections, diagnosis, Telemedicine, Students, Medical, Unified Health System

## Abstract

O presente relato de experiência tem como objetivo descrever a concepção e os dados preliminares da implementação de um programa de telerrastreio e telemonitoramento da covid-19 para usuários do Sistema Único de Saúde com condições de risco para agravamento. Foi implantado um sistema de telerrastreio por meio do qual estudantes de graduação na área da saúde contactam os pacientes via telefone, conforme periodicidade e critérios predefinidos, para monitorar a evolução do quadro. Em oito semanas, foram realizadas 2.190 tentativas de contato remoto com indivíduos de cinco unidades de saúde. O número efetivo de indivíduos monitorados no momento da escrita deste artigo é de 802.

## INTRODUÇÃO

O surgimento do novo coronavírus, denominado Sars-CoV-2, apresenta um desafio sem precedentes para os profissionais de saúde em todo o mundo, provocando impactos globais na saúde, na política e na economia em poucos meses. Em janeiro de 2020, a Organização Mundial da Saúde (OMS) declarou a covid-19 uma emergência de saúde pública de interesse internacional e, em março, sua configuração pandêmica^1^. Não obstante os óbitos relacionados à doença ocorrerem principalmente entre pessoas com idade mais avançada, estudos mais recentes apontam que indivíduos com polimorbidades, como hipertensão, diabetes e doenças cardiovasculares, podem ter mais riscos de complicações pela doença^2^.

Embora venha sendo uma estratégia bastante utilizada para minimizar a propagação da doença, o isolamento social trouxe dificuldades à garantia de atenção integral à saúde dos usuários, inclusive na busca contínua dos casos ativos da covid-19 e dos seus contatos, conforme recomendado pela OMS^3^. Há relatos de que a telemedicina tem sido usada para retardar a disseminação do Sars-CoV-2, mantendo o distanciamento social e fornecendo serviços telefônicos para cuidados em casos leves, mas essa modalidade de assistência apresenta limitações nos casos urgentes^4^.

Em um esforço para manter o rastreio da covid-19, especialmente entre os grupos considerados de risco, e continuar a prestação dos cuidados essenciais aos usuários, o telerrastreio pode ser um mecanismo seguro e crucial para a manutenção dos cuidados, especialmente entre usuários com maior risco, que podem evoluir rapidamente para insuficiência respiratória aguda. Neste artigo, descrevemos a concepção e dados preliminares da implementação de um programa de telerrastreio da covid-19 com usuários do Sistema Único de Saúde (SUS) com condições de risco em Vitória da Conquista, município de médio porte do estado da Bahia, entre 31 de maio e 30 de julho de 2020.

## MÉTODOS

O presente artigo traz um relato de experiência da implantação de um programa de telerrastreio de pacientes com síndrome gripal, pertencentes a grupos de risco da covid-19, cadastrados como usuários de unidades de saúde da família (USF) do município de Vitória da Conquista, Bahia.

O município tem população estimada em 341.597 habitantes, segundo estimativa do Censo IBGE 2019, e é sede da macrorregião do Sudoeste da Bahia, referência para aproximadamente 80 municípios^5^. A atenção primária no município está dividida entre 47 equipes de saúde da família, 7 unidades básicas de saúde e 4 núcleos ampliados de saúde da família e atenção básica, com cobertura populacional de 48% pela Estratégia Saúde da Família, e de 60% pela atenção básica, segundo dados do portal e-Gestor AB, do Ministério da Saúde^6^. A atenção básica do município não utilizava estratégias de telessaúde para atendimento não presencial de usuários do SUS no período anterior à pandemia da covid-19.

Um grupo de trabalho composto por docentes de uma universidade local, profissionais da atenção primária e representantes da Diretoria da Atenção Básica da Secretaria Municipal de Saúde elaborou um fluxo de telerrastreio e telemonitoramento de pacientes com sinais e sintomas de síndrome gripal. Neste fluxo, também foi inserida a etapa de atendimento presencial.

O fluxo foi direcionado para a identificação de pacientes considerados de maior risco para agravamento da covid-19, como idosos, portadores de doenças crônicas ou autoimunes e indivíduos obesos (Figura). Os números de telefone destes usuários foram identificados pelo sistema e-SUS Atenção Primária ou fornecidos pelos agentes comunitários de saúde. Para realizar o telerrastreio e telemonitoramento foram alugadas linhas telefônicas disponibilizadas aos estudantes de graduação da área da saúde.

**Figura:** Fluxograma do telerrastreio da covid-19 em pacientes da atenção primária à saúde que possuem condições de risco para o desenvolvimento de quadro grave, no município de Vitória da Conquista - Bahia.

Os usuários cadastrados nas USF receberam a primeira ligação em horário comercial. Neste primeiro momento, o motivo da ligação foi explicado e coletaram-se dados de identificação do indivíduo. Os dados foram lançados em um sistema na plataforma web para acompanhamento de casos de covid-19, desenvolvido pelo Núcleo de Tecnologia da Informação do município. Esta aplicação funciona como um sistema de suporte à decisão clínica que classifica o usuário como assintomático ou com síndrome gripal leve ou grave, de acordo com os critérios do Ministério da Saúde. Os indivíduos com síndrome gripal leve e que têm polimorbidades são classificados pelo sistema em síndrome gripal moderada, considerando o maior risco de agravamento da covid-19 nestes casos.

Para os casos de síndrome gripal leve ou moderada, as equipes de saúde da família monitoram os usuários a cada 48 horas, e para os casos de síndrome grave a cada 24 horas. Os casos assintomáticos recebem outra ligação telefônica da equipe de telerrastreio em 14 dias. Em todos os casos sintomáticos são realizados RT-PCR ou testes sorológicos pela equipe da Secretaria Municipal de Saúde. Os testes sorológicos são realizados para os casos que não estão na janela de tempo ótima de coleta do material para o RT-PCR. Todos os casos positivos para covid-19 e seus contatos são monitorados pelas equipes da atenção básica, com participação da vigilância epidemiológica do município.

As equipes de saúde e os estudantes envolvidos no telerrastreio e telemonitoramento receberam treinamento para utilizar a plataforma web desenvolvida. A Secretaria Municipal de Saúde atuou na divulgação da ação entre os moradores dos bairros a fim de aumentar a adesão da comunidade no atendimento das ligações. Os dados foram descritos através de medidas de tendência central pertinentes e frequência simples.

## RESULTADOS

Decorridas oito semanas, foram realizadas 2.190 tentativas de contato remoto com os indivíduos. Desse total, o número efetivo de indivíduos monitorados foi de 802 (36,6%), com média de idade de 59,7 anos (DP=18,5), sendo 532 (66,3%) do sexo feminino. Foram identificados 15 (1,6%) indivíduos com síndrome gripal leve ou moderada e 7 (0,7%) com síndrome gripal grave, os quais foram encaminhados para o teleatendimento. A proporção de chamadas telefônicas recusadas foi de 38,9% (853).

Outro aspecto que se destacou foi o percentual de números telefônicos que não estavam atualizados no sistema municipal de saúde: 24,4% (535) tendo como justificativa a não obrigatoriedade do número de telefone para o cadastro municipal nas USF ou a perda da linha devido às condições financeiras dos pacientes. Para superar esses desafios, foram colocadas em prática estratégias que incluíram cartões digitais para divulgação via aplicativo de mensagens, materiais impressos, como fôlderes e cartazes, divulgação nos territórios pelos agentes comunitários de saúde e nas USF pelos profissionais que prestam atendimento presencial. Para os números telefônicos desatualizados, os agentes comunitários fizeram busca ativa dos indivíduos no território e, quando possível, realizaram o rastreio presencialmente.

## DISCUSSÃO

Não foi possível o rastreio da covid-19 em mais da metade dos usuários contactados. Esse dado demonstra como a atenção primária à saúde precisa debater questões referentes ao uso das tecnologias de informação e comunicação dentro da sua rotina de trabalho, entendendo que essa ação ampliará o acesso da comunidade aos serviços de saúde, não apenas no período de pandemia. A atenção primária deve coordenar o cuidado dentro da rede assistencial, consolidando o uso de tecnologias de informação e comunicação e apoiando ações de acompanhamento, vigilância, informação e fortalecimento do cuidado à saúde^7^.

Funcionando como um dispositivo de contato remoto com o paciente para triagem de sintomas através de perguntas específicas, o telerrastreio é uma ferramenta importante para promover acesso seguro aos usuários. Seu objetivo é reduzir o tempo necessário para obter um diagnóstico, para iniciar o tratamento e estabelecer os protocolos de atendimentos necessários, bem como permitir o monitoramento domiciliar dos usuários, evitando a saturação das unidades e o risco de contágio, informando os cidadãos e propondo uma estratégia de comunicação em tempos de distanciamento^4^. Essa estratégia auxilia a atenção primária à saúde com o gerenciamento domiciliar das condições de saúde dos usuários, mantendo o atendimento longitudinal e contínuo.

Foram efetivados muitos contatos remotos em um curto período, o que ajudou a identificar alguns pacientes suspeitos, posteriormente monitorados pelas equipes de saúde das unidades. Nesse momento é possível fornecer aos usuários orientações gerais, além de aumentar a conscientização sobre a pandemia e aliviar o estresse psicológico das comunidades acompanhadas. De qualquer modo, mesmo através de visitas domiciliares por agentes comunitários, seria difícil alcançar todos os pacientes, e há ainda a vantagem de uma provável proteção aos profissionais de saúde, sem contato direto com pacientes suspeitos de covid-19. Ademais, o uso de ferramentas de telessaúde pode ser útil para o atendimento rotineiro da atenção básica no período pós-pandemia. A nova abordagem também teve um custo baixo e mostrou-se útil no acompanhamento dos usuários por agentes comunitários de saúde e até mesmo por outros profissionais das equipes de saúde da família.

Por se tratar de um relato de experiência, os resultados apresentam algumas limitações. A realidade de um município de médio porte pode não ser reproduzível em cidades menores, em que a qualidade do sinal de telefonia móvel ou internet apresente maior instabilidade. Além disso, estudantes de graduação fizeram o telerrastreio.

O telerrastreio demonstrou-se uma ação operacionalmente viável, promotora de comunicação rápida com a comunidade dos territórios, devendo ser fortalecida para o acompanhamento das doenças crônicas não transmissíveis e o apoio à rotina dos serviços. Nesse período de pandemia, o telerrastreio foi importante para o cuidado precoce dos usuários.
